# GLP-1 Receptor Agonists, Gut Microbiota, and Chronic Pain: Nutritional Signaling at the Gut–Brain Axis

**DOI:** 10.3390/nu18193120

**Published:** 2026-09-22

**Authors:** Charles A. Odonkor, Mustafa R. Dodurgali, Fiona R. Cox, Ian J. Klimov, Alaa Abd-Elsayed

**Affiliations:** 1 Department of Orthopaedics & Rehabilitation, Yale School of Medicine, New Haven, CT 06510, USA; mustafa.dodurgali@yale.edu; 2 Neuromodulation and Digital Health Lab, Yale University, New Haven, CT 06510, USA; fionacox601@gmail.com (F.R.C.); ian.klimov@yale.edu (I.J.K.); 3 Department of Anesthesiology, University of Wisconsin–Madison, Madison, WI 53792, USA; abdelsayed@wisc.edu

**Keywords:** gut microbiota, gut–brain axis, GLP-1 receptor agonists, chronic pain, short-chain fatty acids, enteroendocrine cells, neuroinflammation, dysbiosis, nutritional signaling, nociception

## Abstract

Background/Objectives: Chronic pain affects more than one in five adults worldwide and is increasingly conceptualized as a disorder of the gut–brain axis. Gut microbial dysbiosis, characterized by reduced alpha-diversity and depletion of short-chain fatty acid (SCFA)-producing taxa such as *Faecalibacterium prausnitzii*, *Lachnospiraceae*, and *Roseburia*, has been mechanistically linked to pain sensitization. Glucagon-like peptide-1 (GLP-1), a nutrient-sensing enteroendocrine hormone, and its pharmacological receptor agonists (GLP-1RAs) both reshape the gut microbiota and modulate pain across multiple phenotypes. This review synthesizes preclinical and clinical evidence positioning the gut microbiota as a proposed nutritional intermediary that links GLP-1RA pharmacology to chronic pain modulation. Methods: We conducted a narrative synthesis of studies retrieved from PubMed, Embase, and Web of Science from inception through 30 June 2026, spanning GLP-1 nutrient sensing, gut microbial alterations in pain, GLP-1RA effects on the microbiome, microbiota-dependent mechanisms, and clinical pain outcomes. Results: GLP-1 is secreted by L-cells in response to nutrients and microbial SCFAs, signaling to the brain through vagal and humoral routes. GLP-1RAs enrich the same anti-inflammatory, SCFA-producing taxa that are depleted in chronic pain, and their anti-inflammatory actions require an intact microbiota. Mechanistically, GLP-1RAs suppress pain through spinal microglial GLP-1 receptor (GLP-1R)/interleukin-10/beta-endorphin signaling, Nucleotide-binding oligomerization domain (NOD)-, Leucine-rich repeat (LRR)-, and pyrin domain-containing protein 3 (NLRP3) inflammasome inhibition, and receptor-independent transient receptor potential vanilloid 1 (TRPV1) blockade, largely independent of glycemic control or weight loss. Clinically, evidence is strongest for knee osteoarthritis and idiopathic intracranial hypertension headache, with emerging signals in fibromyalgia, visceral pain, and reduced opioid use. Conclusions: The gut microbiota is proposed to act as both a therapeutic target and a mechanistic intermediary in GLP-1RA-mediated analgesia, a model that awaits direct confirmation in humans. Pain-specific trials incorporating dietary and microbiome endpoints are needed.

## 1. Introduction

Chronic pain affects more than 20% of adults globally and remains one of the leading causes of disability and health-care expenditure. Conventional analgesics (nonsteroidal anti-inflammatory drugs, anticonvulsants, antidepressants, and opioids) offer incomplete relief and carry substantial adverse effects and dependence liabilities. This therapeutic gap has motivated a search for novel mechanistic interventions, and one of the most unexpected candidates has emerged from metabolic medicine: the glucagon-like peptide-1 receptor agonists (GLP-1RAs) [1,2].

The scale of the problem is considerable. Chronic pain is among the most common reasons adults seek medical care, a leading contributor to years lived with disability, and a principal driver of long-term opioid exposure. The pharmacological options that dominate practice were largely designed to interrupt neural signaling rather than to address the systemic and metabolic-inflammatory context in which pain is generated and maintained. As appreciation has grown for the roles of neuroinflammation, glial activation, and immune-neural crosstalk in the transition from acute to chronic pain, so too has interest in interventions targeting these upstream processes. This includes the gut’s metabolic-inflammatory milieu, which is increasingly gaining prominence.

In parallel, the conceptual framing of chronic pain has shifted. Pain is no longer understood as a purely neural phenomenon but as an emergent property of a bidirectional gut–brain axis in which the intestinal microbiota, the enteroendocrine epithelium, the enteric and vagal nervous systems, and central pain circuits are continuously in dialogue [3]. A systematic review and meta-analysis of patients with heterogeneous chronic pain conditions found consistent reductions in microbial alpha-diversity and depletion of short-chain fatty acid (SCFA)-producing taxa (*Faecalibacterium prausnitzii*, *Lachnospiraceae*, and *Roseburia*) relative to pain-free controls [4]. Because these taxa are strongly shaped by diet, particularly by the intake of fermentable fiber, the gut–brain axis provides a plausible route through which nutrition influences nociception [5,6]. 

GLP-1 sits precisely at this nutritional interface. It is an incretin hormone secreted by enteroendocrine L-cells in direct response to ingested and microbially derived nutrients, and it signals to the brain through both humoral and vagal routes [7]. GLP-1RAs, developed for type 2 diabetes and obesity, are long-acting pharmacological mimics of this endogenous nutrient signal. A growing body of literature demonstrates that, beyond their metabolic effects, GLP-1RAs remodel the gut microbiota and exert analgesic effects across diverse pain phenotypes [2,8,9].

This review advances a single organizing thesis: that the gut microbiota is a central nutritional intermediary linking GLP-1RA pharmacology to chronic pain modulation. We first situate GLP-1 within nutrient-sensing enteroendocrine physiology; map the distribution of GLP-1 receptors (GLP-1R) along the pain neuraxis; and trace the vagal, humoral, and neuroimmune routes connecting the gut to pain circuits. We then examine the microbiota as a mechanistic mediator, integrating evidence that GLP-1RAs enrich the very taxa depleted in chronic pain and that microbial SCFAs act as signaling molecules in nociception. Finally, we summarize preclinical mechanisms and clinical evidence by pain condition, address the central question of weight-dependent versus weight-independent analgesia, and then outline the nutritional and translational implications of viewing GLP-1RAs as microbiota-modulating agents.

## 2. Literature Search Strategy

This is a comprehensive narrative review. We searched PubMed, Embase, and Web of Science for literature published from inception through 30 June 2026 using combinations of the terms “GLP-1”, “glucagon-like peptide-1”, “incretin”, “gut microbiota”, “short-chain fatty acids”, “enteroendocrine”, “gut–brain axis”, “chronic pain”, “nociception”, “neuropathic pain”, “osteoarthritis”, “fibromyalgia”, and “visceral pain”. Studies were considered relevant if they addressed one or more of the following domains: GLP-1 nutrient-sensing physiology; gut microbial alterations in chronic pain states; effects of GLP-1RAs on microbiome composition; microbiota-dependent mechanisms of GLP-1RA action; or clinical pain outcomes with GLP-1RA therapy. Preclinical mechanistic studies, randomized controlled trials, systematic reviews and meta-analyses, and large observational cohorts were all considered. Because the aim of this article is integrative synthesis rather than quantitative pooling, we did not perform a formal risk-of-bias assessment or a quantitative meta-analysis. The focused eligibility search combining GLP-1 terms with pain terms identified 825 records in total—PubMed/MEDLINE [*n* = 243], Embase [*n* = 250], and Web of Science [*n* = 332] (searches to 30 June 2026; the study-selection flow is shown in Figure 1, and the full per-database strings and eligibility criteria are compiled in File S1); after the removal of 323 duplicate records, the remaining 502 unique records were screened by title and abstract against the domains listed above; 300 records were excluded at this stage, and of the 202 full-text reports subsequently assessed for eligibility, 122 were excluded and 80 studies met the eligibility criteria. Throughout, we prioritized peer-reviewed primary studies, randomized controlled trials, systematic reviews and meta-analyses, and large observational or propensity-matched cohorts, supplemented by seminal earlier work and additional articles identified by hand-searching reference lists. Records were excluded if they did not address GLP-1 or GLP-1 receptor agonist biology, gut microbial signaling, or a pain-relevant outcome, or if they were available only as conference abstracts or as non-English publications without an available translation. The synthesis ultimately draws on 97 references, comprising studies that met these criteria together with foundational and contextual works identified from the reference lists of key articles.

## 3. GLP-1 as a Nutrient-Sensing Signal: From L-Cells to the Nervous System

The enteroendocrine epithelium is the largest sensory surface in the body. Scattered among absorptive enterocytes, hormone-secreting enteroendocrine cells sample the luminal contents and translate nutrient and microbial signals into circulating and paracrine messengers [7]. Among these, specialized, large electron-dense secretory cell granules, known as L-cells, are concentrated in the distal small intestine and colon. They serve as the gut’s primary nutrient sensors and secrete GLP-1 in response to the digestion products of carbohydrates, proteins, and lipids, as well as microbial metabolites. In healthy adults, circulating total GLP-1 rises from a fasting concentration of approximately 5 to 15 pmol/L to postprandial peaks roughly two- to threefold higher (on the order of 20 to 50 pmol/L), although the biologically active, intact fraction is considerably lower because most secreted GLP-1 is rapidly degraded by dipeptidyl peptidase-4 (DPP-4) within minutes of release [10,11]. GLP-1 secretion is thus not a fixed endocrine reflex but a continuous readout of diet and of the microbial community that ferments it. Once released, GLP-1 communicates the presence of food to multiple organs: (a) the pancreas (stimulates glucose-dependent insulin secretion and suppresses glucagon), (b) the stomach (delays gastric emptying), (c) the brain (promotes satiety and modulates autonomic and pain-processing circuits), (d) the vagus nerve (activates vagal afferent fibers, transmitting nutrient information from the gut to the brainstem), and (e) the immune system (reduces inflammation and helps maintain intestinal barrier integrity). Thus, GLP-1 functions as an endocrine, paracrine, and neurocrine signal to coordinate metabolism, gastrointestinal function, immune responses, and communication between the gut and the nervous system.

A particularly important class of L-cell stimuli is the SCFAs (acetate, propionate, and butyrate) produced by bacterial fermentation of dietary fiber in the colon. SCFAs stimulate GLP-1 secretion through the G-protein-coupled free fatty acid receptors FFAR2 (GPR43) and FFAR3 (GPR41): in primary intestinal cultures and in vivo, SCFA-evoked GLP-1 release is markedly attenuated in *Ffar2*-deficient mice, establishing FFAR2 as a required receptor [12]. Propionate in particular acutely stimulates GLP-1 and peptide YY secretion through FFAR2 [13]. This receptor-level link means that the amount of GLP-1 an individual secretes is partly determined by the fermentative output of their microbiota, and therefore by dietary fiber intake.

Human studies confirm the nutritional relevance of this pathway. Targeted delivery of propionate to the human colon, using an inulin-propionate ester, significantly increased postprandial plasma GLP-1 and peptide YY and reduced weight gain in overweight adults [14]. Similarly, fermentation of the prebiotic oligofructose raised plasma GLP-1, enhanced satiety, and improved postprandial glucose handling [15]. Endogenous GLP-1 secretion is therefore a modifiable, diet- and microbiota-dependent signal.

This nutrient-sensing framing has an important corollary for pain research. The same dietary and microbial factors that determine metabolic health (fiber intake, fermentative capacity, and the abundance of SCFA-producing taxa) also set the tone of endogenous GLP-1 secretion. If GLP-1 signaling contributes to nociceptive regulation, then diet and the gut microbiota are not merely background variables but upstream determinants of an analgesic axis. This positions nutrition as a modifiable lever on a pain-relevant hormonal system, and distinguishes the present synthesis from accounts that treat GLP-1RAs purely as pharmacological agents divorced from their physiological, nutrient-driven context.

Once released, GLP-1 acts through two parallel routes. In the endocrine route, intact GLP-1 that survives degradation reaches target tissues via the bloodstream; however, roughly 90% of secreted GLP-1 is inactivated by dipeptidyl peptidase-4 (DPP-4) within minutes of release [10]. In the neural route, GLP-1R on vagal afferent terminals located near L-cells is activated in a paracrine fashion before degradation, thereby relaying nutrient information to the brainstem [16,17]. This distinction is pharmacologically consequential: subcutaneously injected GLP-1RAs are resistant to DPP-4 and reach target cells predominantly through the systemic circulation at sustained, supraphysiological concentrations, potentially engaging GLP-1R populations that endogenous, rapidly degraded GLP-1 rarely reaches [17]. Endogenous nutrient-driven GLP-1 and pharmacological GLP-1RAs thus overlap but are not identical signals, a theme that recurs throughout this review.

## 4. GLP-1 Receptor Distribution Across the Pain Neuraxis

Interpreting the analgesic actions of GLP-1RAs requires knowing where GLP-1R is expressed. Anatomical and functional studies place GLP-1R at every level of the pain neuraxis, from the gut wall to the cortex (Figure 2).

In the periphery, GLP-1R immunoreactivity is intense in small- and medium-diameter neurons of human dorsal root ganglia (DRG) and in cultured human and rodent DRG neurons [18]. GLP-1R has been confirmed in lumbar DRG, sciatic nerve, and skin of both diabetic and nondiabetic mice [19,20,21]. Within the gut, GLP-1R is expressed on colonic mucosal nerve fibers, with a significantly increased number of GLP-1R-positive fibers in inflammatory bowel disease biopsies (*p* = 0.0013), and on enteric, extrinsic spinal, and vagal sensory afferents [18,22]. This gut-localized receptor expression is the anatomical substrate for a direct enteric contribution to GLP-1-mediated sensory modulation.

In the spinal cord, GLP-1R is expressed by dorsal horn microglia and is markedly upregulated after peripheral nerve injury; GLP-1 and GLP-1R proteins are also detectable in neurons, astrocytes, and microglia, with particular abundance in Iba1-positive microglia [23,24]. Brainstem preproglucagon (PPG) neurons in the nucleus tractus solitarius (NTS) send varicose axons to the deep dorsal horn of the lower lumbar cord, thereby providing an endogenous source of spinal GLP-1 that positions the peptide to modulate incoming nociceptive traffic [25]. Supraspinally, GLP-1R is highly expressed in several brain regions, including: the NTS, area postrema, hypothalamus, interpeduncular and tegmental nuclei, lateral septum, and nucleus accumbens. In these regions, GLP-1R colocalizes predominantly with GABAergic neurons; the one exception is in the hippocampus, where it associates with glutamatergic neurons. Subsequently, GLP-1R is upregulated on reactive astrocytes and microglia during inflammation [26]. Functional imaging supports the relevance of these sites for pain: positron-emission tomography has confirmed microglial activation in the cortex and thalamus of diabetic neuropathic-pain models, which GLP-1RA administration attenuates [27]. This distribution, which is enriched at precisely the sites that generate, relay, and amplify pain, frames GLP-1R as a target that can be engaged throughout the neuraxis rather than at a single locus.

## 5. The Gut–Brain Axis: Signaling Routes from Gut to Pain Circuits

Two broad routes carry GLP-1 signals from the gut to central pain circuits, and their relative engagement differs between endogenous GLP-1 and pharmacological GLP-1RAs (Figure 3).

### 5.1. Vagal Afferent Pathway

Enteroendocrine L-cells release GLP-1 postprandially, but because DPP-4 degrades most of it before it reaches the circulation, a major share of its signaling occurs locally, through GLP-1R on glutamatergic vagal afferent terminals near L-cells [16,26]. These afferents relay to the NTS, where a population of GABAergic PPG neurons synthesize and release GLP-1 centrally in response to vagal input, gastric distension, and humoral signals. The same afferents project widely to central autonomic control regions, mesolimbic reward centers, and spinal autonomic neurons [26,28]. This architecture means that a meal, its fiber content, and the microbial fermentation it supports can, through vagal and PPG relays, influence circuits that set both the sensory and the affective dimensions of pain. The relevance of vagal gut–brain signaling to pain is now concrete: a glutamatergic tetra-synaptic NTS-to-parabrachial-to-paraventricular-thalamic-to-prelimbic pathway is necessary and sufficient for gastric pain processing. It is driven primarily by the vagus nerve [29] and a separate caudal-NTS-to-periaqueductal-gray pathway mediates vagal suppression of somatic pain and negative affect [30]. Nutrient- and microbe-evoked GLP-1 secretion can, in principle, tune all of these circuits.

### 5.2. Systemic and Direct Central Engagement

Subcutaneously injected GLP-1RAs reach targets mainly via the systemic circulation, potentially bypassing the vagal paracrine route [17]. Although circulating GLP-1 crosses the blood–brain barrier by diffusion, only a small fraction of peripherally secreted GLP-1 reaches the central nervous system, reinforcing the idea that endogenous and pharmacological ligands activate partly distinct receptor pools [26]. Notably, semaglutide prevents neutrophil infiltration into the brain. It suppresses neuroinflammation-associated transcriptional signatures specifically in microglia, endothelial cells, and pericytes, with a subset of *Glp1r*-expressing neurons in the dorsal vagal complex regulating anti-inflammatory gene expression [31].

### 5.3. Neuroimmune Axis: Central GLP-1R Drives Peripheral Anti-Inflammation

A pivotal study showed that GLP-1R activation attenuates systemic tumor necrosis factor-α (TNF-α) induction by multiple Toll-like receptor agonists, and that this effect requires central neuronal GLP-1R, not hematopoietic or endothelial receptors, acting through alpha1-adrenergic, delta-opioid, and kappa-opioid signaling [32]. This establishes a gut–brain GLP-1R axis that suppresses peripheral inflammation, linking a nutrient-sensing hormone to systemic immune tone and, by extension, to inflammatory pain.

## 6. The Gut Microbiota as the Mechanistic Intermediary

If GLP-1 is the nutrient signal and the neuraxis is the target, the gut microbiota is the medium in which the two are coupled (Figure 4). This section develops the review’s central argument in four steps: (1) chronic pain is associated with a characteristic dysbiosis; (2) GLP-1RAs reverse that dysbiosis; (3) microbial SCFAs act as signaling molecules relevant to pain; and (4) enteroendocrine cells translate the microbial–nutritional milieu into nociceptor activity.

### 6.1. Microbiome Alterations in Chronic Pain

Across chronic pain conditions, a systematic review and meta-analysis (21 studies) found reduced alpha-diversity in patients versus controls: lower observed species (standardized mean difference [SMD] = −0.20; *p* = 0.01), Shannon index (SMD = −0.27; *p* < 0.001), and Faith phylogenetic diversity (SMD = −0.35; *p* = 0.01), together with decreased relative abundance of *Lachnospiraceae*, *Faecalibacterium*, *Roseburia*, and *F. prausnitzii* and increased *Eggerthella* [4]. Because *F. prausnitzii* is a prototypical anti-inflammatory commensal whose depletion tracks with intestinal and systemic inflammation, its loss is mechanistically salient rather than incidental [33].

The strongest causal evidence comes from fibromyalgia. Fecal microbiota transplantation from patients with fibromyalgia into germ-free mice induced pain and molecular phenotypes paralleling the human disease, including immune activation and metabolomic alterations. In contrast, replacing the fibromyalgia microbiota with a healthy community substantially alleviated pain; an open-label transplantation trial in affected women reported reduced pain and improved quality of life [34]. Earlier work had already established a distinctive fibromyalgia-associated microbial signature, with taxonomic changes correlating with symptom severity [35]. Beyond fibromyalgia, nerve injury itself remodels the microbiome: injury-induced gut dysbiosis contributes to spinal cord TNF-α expression and nociceptive sensitization [36]. Subdiaphragmatic vagotomy blocks the onset of complete Freund’s adjuvant-induced chronic inflammatory pain and neuroinflammation. This demonstrates that the gut–microbiota–brain axis operating through the vagus nerve is required for chronic pain to develop [37].

A complementary strand concerns the intestinal barrier. Dysbiosis and depletion of butyrate-producing taxa compromise epithelial tight junctions, permitting translocation of microbial products such as lipopolysaccharides, which raise systemic inflammatory tone and can sensitize nociceptors. GLP-1RAs also act at this interface. In preclinical models, liraglutide improves gut barrier integrity and reduces visceral hypersensitivity [38]. GLP-1RA therapy also enriches barrier-supporting commensals, particularly *Akkermansia muciniphila*, which independently strengthen the intestinal epithelium and reduce metabolic inflammation [39,40]. A more intact barrier and a less inflammatory lumen together reduce the peripheral drive that maintains chronic pain, linking the microbial findings to the neuroimmune mechanisms detailed in Section 7.

### 6.2. GLP-1RAs Reshape the Microbiome

GLP-1RAs act on the same microbial community that is disrupted in chronic pain, and they shift it in the opposite direction. In diabetic rats, liraglutide substantially restructured the gut microbiota, enriching SCFA-producing taxa (*Bacteroides*, *Lachnospiraceae*) and probiotic *Bifidobacterium* [41]. In db/db mice liraglutide increased *A. muciniphila* while attenuating hepatic steatosis [42]. *A. muciniphila* is itself a mucin-associated commensal that reinforces gut-barrier integrity and improves metabolic-inflammatory status in both mice and humans [39,40]. Contemporary reviews now list beneficial modulation of the intestinal microbiome as a recognized mechanism of GLP-1RA action [8,43].

Two findings tie these microbial shifts to the drug’s therapeutic effects. First, the gut intraepithelial-lymphocyte GLP-1R is required for the full effects of GLP-1RAs on microbiota composition, and this control is dissociable from glucose and weight regulation [44]. Second, GLP-1RAs require an intact microbiota for their anti-inflammatory action: in colitis models, antibiotic depletion abolishes therapeutic benefit, and GLP-1RA treatment modulates group 3 innate lymphoid cell function through the microbiota [45]. Clinically, in a small study of patients with type 2 diabetes, GLP-1RA therapy significantly increased *F. prausnitzii* abundance (*p* = 0.002), a taxon that is anti-inflammatory and found to be depleted in chronic pain states [4,46]. Taken together, these observations are consistent with the possibility that GLP-1RAs enrich several of the same taxa that are depleted in chronic pain, and that their microbial effects are at least partly separable from glucose and weight control. It should be emphasized, however, that these data derive largely from preclinical models and small clinical studies, and that no study has yet demonstrated in humans that GLP-1RA-induced microbial shifts causally mediate analgesia.

The mechanistic thread linking these observations is the dependence of GLP-1RA immunomodulation on the intestinal ecosystem. The gut intraepithelial-lymphocyte GLP-1R and group 3 innate lymphoid cells identify specific cellular relays through which luminal microbial signals are converted into mucosal and systemic immune tone [44,45]. When these relays are disrupted, whether genetically or by antibiotic depletion of the microbiota, the drug loses part of its therapeutic effect, demonstrating that the microbiota is not downstream scenery but an obligatory participant. In parallel, taxa such as *A. muciniphila* and *F. prausnitzii* contribute anti-inflammatory and barrier-protective functions that plausibly reduce the tonic inflammatory input reaching nociceptors [33,39]. Collectively, these findings are compatible with a model in which GLP-1RAs act, in part, by favoring a less inflammatory microbial community; this model rests mainly on preclinical data, and direct evidence that microbiota-mediated changes drive analgesia in patients is currently lacking.

### 6.3. SCFAs and Microbial Metabolites as Nutritional Signaling Molecules

SCFAs are the principal currency through which diet and the microbiota communicate with the host, and they are increasingly implicated in nociception. Produced by bacterial fermentation of otherwise indigestible dietary fiber, acetate, propionate, and butyrate reach millimolar concentrations in the colonic lumen, where they nourish the epithelium, regulate tight-junction integrity, shape mucosal and systemic immune tone, and engage FFAR2/FFAR3 on enteroendocrine and immune cells [47,48]. Through these actions they signal via the endocrine, vagal, immune, and humoral routes of the microbiota–gut–brain axis, ultimately influencing central nervous system activity [3,47].

The relevance of SCFAs to pain is both indirect, through barrier and immune effects that lower the inflammatory drive to nociceptors, and direct. In rat models of irritable bowel syndrome, butyrate inhibited visceral allodynia and reduced colonic hyperpermeability [49]. In healthy volunteers, colonic butyrate enemas modulated visceral perception, providing human evidence that a microbial metabolite can alter nociceptive signaling [50]. The unifying observation of this review is that SCFAs sit at a triple intersection: they are products of a fiber-rich diet, they stimulate L-cell GLP-1 secretion through FFAR2 [12,13], and they modulate pain. A fiber-rich diet, an endogenous incretin response, a GLP-1RA, and analgesia therefore share a common metabolite node, which is precisely why the microbiota is better viewed as a mechanistic intermediary than as a passive marker.

### 6.4. Enteroendocrine Cell-Nociceptor Crosstalk

The enteroendocrine epithelium not only secretes hormones but also communicates directly with sensory nerves. Enterochromaffin cells synthesize roughly 95% of the body’s serotonin and detect noxious and mechanical stimuli, forming synaptic-like contacts with 5-HT3-receptor-expressing sensory afferents [51,52]. These cells are both necessary and sufficient for visceral pain. Enhancing enterochromaffin cell function elicits hypersensitivity to gut distension, whereas suppressing it blocks the sensitizing effects of bacterial SCFAs. Prolonged enterochromaffin cell activation produces persistent visceral hypersensitivity even without an inflammatory trigger [53]. Incretins participate directly in this crosstalk: GLP-1 and glucose-dependent insulinotropic polypeptide (GIP) stimulate substance P release from transient receptor potential vanilloid 1 (TRPV1)- and Transient Receptor Potential Ankyrin 1 (TRPA1)-expressing sensory nerves, underscoring a previously unrecognized neurolymphocrine action of incretins on primary afferents [22]. The same enterochromaffin-cell serotonin system is a target of GLP-1RA action elsewhere in the gut: exendin-4 reduces colonic serotonin, upregulates the serotonin transporter, and downregulates tryptophan hydroxylase-1. This is consistent with modulation of serotonergic tone in visceral pain [54]. Enteroendocrine cells thus constitute a bidirectional interface where microbial metabolites, incretin signaling, serotonergic transmission, and nociceptor excitability converge. This interface is simultaneously nutritional (it senses the products of digestion and fermentation) and nociceptive (it wires to pain-carrying afferents). It is here, at the epithelial surface, that the review’s two central actors, the nutrient-sensing incretin system and the pain-relevant sensory apparatus, are most intimately joined.

## 7. Preclinical Mechanisms of GLP-1RA Analgesia

At least ten mechanisms of GLP-1RA-mediated analgesia have been characterized, operating at peripheral, spinal, and supraspinal levels (Table 1). A unifying feature is that they suppress pathological pain hypersensitivity (allodynia and hyperalgesia) while largely sparing acute nociception; they also operate independently of glycemic control and weight loss.

Mechanistically, SCFA binding to FFAR2/FFAR3 couples through Gq/11 and Gi/o proteins to mobilize intracellular Ca2+ and, together with closure of ATP-sensitive and other K+ channels, depolarizes the L-cell membrane; the resulting voltage-gated Ca2+ entry triggers Ca2+-dependent exocytosis of GLP-1-containing secretory granules (Figure 5A) [12,13]. The best-characterized mechanism is the spinal microglial GLP-1R/beta-endorphin pathway (Figure 5B). Intrathecal GLP-1R agonists alleviate nerve injury-, formalin-, bone cancer-, and diabetes-induced hypersensitivity by 60–90% without affecting acute nociceptive responses [23]. The cascade proceeds from GLP-1R through cAMP/PKA/p38beta/CREB to autocrine interleukin-10 (IL-10), which acts on the IL-10 receptor-alpha/STAT3 pathway to drive beta-endorphin expression. Exenatide stimulates microglial proopiomelanocortin and beta-endorphin with EC50 values in the low-nanomolar range. Antinociception is abolished by the microglial inhibitor minocycline, beta-endorphin antiserum, and naloxone [55,56]. This pathway also restrains central sensitization: exenatide suppresses pathologically enhanced excitatory synaptic transmission in lamina II dorsal horn neurons via the IL-10/beta-endorphin/mu-opioid receptor cascade, without affecting inhibitory transmission [57].

Several features make this pathway attractive as an analgesic mechanism. The antinociception is specific to pathological hypersensitivity, leaving acute protective nociception intact. It is prevented by GLP-1R antagonism and GLP-1R gene knockdown, confirming receptor dependence; and, unlike opioids, repeated dosing does not induce antinociceptive tolerance [23]. Moreover, exenatide stimulates M2-type anti-inflammatory microglial markers (IL-10, IL-4, Arg1, CD206) rather than M1 pro-inflammatory markers. This indicates that the drug reprograms the neuroimmune response toward resolution rather than simply blunting neurotransmission [55]. This resolution-oriented profile mirrors the anti-inflammatory shifts in microbial composition and metabolites described in Section 6, reinforcing the theme that GLP-1RA analgesia is fundamentally an immunomodulatory phenomenon.

GLP-1RAs additionally suppress the Nucleotide-binding oligomerization domain (NOD)-, Leucine-rich repeat (LRR)-, and pyrin domain-containing protein 3 (NLRP3) inflammasome in the brain and cortical microglia, via Glycogen Synthase Kinase-3β (GSK3β)-, AMP-activated protein kinase (AMPK)/sirtuin 1 (SIRT1)-, and nuclear factor erythroid 2-related factor 2 (Nrf2)-dependent pathways, thereby attenuating thermal and mechanical allodynia in diabetic neuropathic pain [27,58,59]. They also inhibit nuclear factor kappa B (NF-κB) and mitogen-activated protein kinase (MAPK) signaling. This reduces the expression of the pro-inflammatory cytokines interleukin-1β (IL-1β) and TNF-α in the hippocampus and peripheral nerves, independently of glycemic control [60,61].

A mechanistically distinct and pharmacologically appealing action is GLP-1R-independent direct inhibition of TRPV1. GLP-1-derived peptides, including the fragment exendin 20–29, directly inhibit capsaicin-evoked TRPV1 currents and relieve pain without engaging GLP-1R or disturbing thermoregulation. This raises the prospect of analgesics without metabolic effects [62]. Further mechanisms include serotonergic modulation [54,63], nitric oxide-dependent visceral analgesia and improved gut permeability [38], neurotrophic support of sensory neurons [19,20,21], blood–brain barrier stabilization [64], and a shift in joint macrophages from a pro-inflammatory M1 to a reparative M2 phenotype in osteoarthritis [65]. Notably, repeated dosing of spinal GLP-1R agonists does not induce antinociceptive tolerance [66].

**Table 1:** Preclinical mechanisms of GLP-1 receptor agonist (GLP-1RA) analgesia across the pain neuraxis.

Mechanism	Level	Key Evidence/Model	Effect	Wt/Gluc. Indep.
Spinal microglial GLP-1R/IL-10/β-endorphin	Spinal	Intrathecal GLP-1R agonists; cAMP/PKA/p38β → IL-10 → β-endorphin [23,55]	60–90% ↓ hypersensitivity	Yes
Suppression of central sensitization	Spinal	↓ enhanced lamina II excitatory transmission via IL-10/β-endorphin/MOR [57]	Normalizes synaptic plasticity	Yes
NLRP3 inflammasome suppression	Central/spinal	GSK3β, AMPK/SIRT1, Nrf2 in microglia [27,58]	↓ thermal/mechanical allodynia	Yes
NF-κB/MAPK inhibition	Central/peripheral	↓ IL-1β, TNF-α in hippocampus and sciatic nerve [60,61]	Anti-inflammatory	Yes
Direct TRPV1 inhibition (GLP-1R-independent)	Peripheral	Exendin 20–29 blocks capsaicin TRPV1 currents [62]	Analgesia without metabolic effect	Yes
Serotonergic modulation	Peripheral/central	5-HT3/opioid dependence; SERT/TPH-1 changes [54,63]	↓ hyperalgesia, visceral pain	Yes
Nitric oxide-dependent visceral analgesia	Gut/visceral	L-NAME-sensitive; improves gut permeability [38]	↓ visceral allodynia	Yes
Neurotrophic/neuroprotective	Peripheral	Neurite outgrowth, Schwann-cell survival [19,20]	Nerve repair in diabetic models	Partly
Blood–brain barrier stabilization	CNS vasculature	↑ occludin/claudin-5 via cAMP/PKA [64]	↓ permeability, neuroprotection	Yes
Joint macrophage M1→M2 polarization	Joint	Intra-articular liraglutide [65]	↓ IL-6, PGE2; anti-degradative	Yes

*Abbreviations:* 5-HT3, 5-hydroxytryptamine (serotonin) receptor 3; AMPK, AMP-activated protein kinase; cAMP, cyclic adenosine monophosphate; CNS, central nervous system; GLP-1R, glucagon-like peptide-1 receptor; GLP-1RA, GLP-1 receptor agonist; GSK3β, glycogen synthase kinase-3β; IL, interleukin; L-NAME, Nω-nitro-L-arginine methyl ester; MAPK, mitogen-activated protein kinase; MOR, μ-opioid receptor; NF-κB, nuclear factor κB; NLRP3, NOD-, LRR-, and pyrin domain-containing protein 3; Nrf2, nuclear factor erythroid 2-related factor 2; PGE2, prostaglandin E2; PKA, protein kinase A; SERT, serotonin transporter; SIRT1, sirtuin 1; TNF-α, tumor necrosis factor-α; TPH-1, tryptophan hydroxylase-1; TRPV1, transient receptor potential vanilloid 1. Wt/gluc. indep., weight- and glucose-independent, i.e., whether the analgesic mechanism has been shown to operate independently of weight loss and glycemic control; in this column, “Yes” denotes demonstrated independence and “Partly” denotes that independence has been shown for some but not all readouts of that mechanism.

## 8. Clinical Evidence Across Chronic Pain Phenotypes

Clinical evidence is uneven across pain conditions, ranging from phase 3 randomized trials to case series (Table 2). We summarize it by phenotype.

### 8.1. Knee Osteoarthritis

The strongest evidence is in knee osteoarthritis, a disease in which mechanical loading, low-grade metabolic inflammation, and central sensitization all contribute to pain [67]. In the STEP 9 trial (*N* = 407; 68 weeks; double-blind, placebo-controlled), once-weekly semaglutide 2.4 mg was superior to placebo on both co-primary endpoints: the Western Ontario and McMaster Universities Osteoarthritis Index (WOMAC) pain score improved by 41.7 points versus 27.5 points (between-group difference −14.2 points; *p* < 0.001), and body weight fell by 13.7% versus 3.2% (*p* < 0.001) [68]. Secondary outcomes moved in concert: 36-Item Short Form Health Survey (SF-36) physical-function score improved by 12.0 versus 6.5 points and 6 min walk distance by an additional 42.6 m, and analgesic use decreased more in the semaglutide arm, confirming a nonsteroidal anti-inflammatory drug-sparing effect [68].

By contrast, a randomized trial of liraglutide 3 mg/day in knee osteoarthritis (*N* = 156) produced significant weight loss (−2.8 kg versus +1.2 kg; *p* = 0.008) but no improvement in Knee injury and Osteoarthritis Outcome Score (KOOS) pain (group difference 0.9 points; *p* = 0.71) [69]. The most economical explanation for the divergence is the magnitude of weight loss: STEP 9 achieved roughly 14% weight loss, whereas the liraglutide trial achieved only a few percentage points [67,68]. Observational and mechanistic data extend the signal beyond weight. A nationwide population-based cohort linked GLP-1RA therapy to lower risk of incident knee osteoarthritis (adjusted hazard ratio [HR] 0.85; *p* < 0.001) and total knee replacement (adjusted HR 0.91; *p* = 0.015) [70]; a large retrospective database analysis found reduced arthroplasty risk over multi-year follow-up [71]; and preclinical work demonstrates weight-loss-independent chondroprotection via a metabolic-restoration mechanism and a shift in synovial macrophages toward a reparative phenotype [65,72].

### 8.2. Idiopathic Intracranial Hypertension and Headache

Idiopathic intracranial hypertension provides some of the clearest evidence for a weight-independent analgesic action. In a phase 2 randomized trial, exenatide significantly lowered intracranial pressure at 2.5 h (−5.7 cmCSF; *p* = 0.048), 24 h (−6.4 cmCSF; *p* = 0.030), and 12 weeks (−5.6 cmCSF; *p* = 0.058), with no change in body mass index over the treatment period, implicating a direct pressure-lowering mechanism, plausibly through GLP-1R at the choroid plexus, rather than weight loss [73]. An open-label study reported that GLP-1RA users had a significantly greater reduction in monthly headache days and a higher 50% headache-responder rate than non-users [74]. Multiple independent meta-analyses converge on reductions in monthly headache days, papilledema, and visual disturbance with GLP-1RA therapy in this population [75,76,77,78]. Because idiopathic intracranial hypertension is itself strongly associated with obesity and recent weight gain, the dissociation of pressure and headache benefit from measurable weight change in the randomized data is mechanistically instructive.

### 8.3. Diabetic Peripheral Neuropathy

Evidence in diabetic peripheral neuropathy concerns nerve structure and function more than pain per se, and it is mixed. A meta-analysis of six studies (*N* = 271) found that GLP-1RA therapy significantly improved nerve conduction velocity compared with controls (mean difference 1.74; 95% confidence interval (CI) 1.16–2.33; *p* < 0.001), independent of changes in blood glucose [79]. A prospective study reported reversal of nerve morphological abnormalities, including improved tibial nerve size, in a majority of participants [80]. However, an 18-month open-label randomized study comparing exenatide with insulin glargine found no significant differences in confirmed clinical neuropathy, intraepidermal nerve fiber density, or nerve conduction [81]. A systematic review concluded that no consistent effect on diabetic peripheral neuropathy has yet been demonstrated in clinical trials [82]. Most studies assessed nerve function or structure rather than pain as a primary endpoint, leaving the analgesic question in this indication genuinely open.

### 8.4. Fibromyalgia and Visceral Pain

In fibromyalgia, where the causal evidence for the microbiome is the strongest (Section 6.1), no dedicated randomized trials exist. Still, a large propensity-matched real-world cohort found that GLP-1RA use was associated with reduced odds of opioid prescription, fewer pain diagnostic codes, and fewer fatigue diagnoses [83]. Body mass index and glycated hemoglobin remained higher in GLP-1RA users, suggesting that benefit was not simply a consequence of metabolic normalization [83]. The juxtaposition is notable: the condition with the clearest evidence that gut microbiota causally drive pain is also one in which a microbiota-modulating drug class shows a real-world analgesic and opioid-sparing signal.

For visceral pain, a systematic review and meta-analysis supports symptomatic improvement of irritable bowel syndrome with GLP-1RAs [84]. The most direct evidence comes from ROSE-010, a GLP-1 analog developed specifically for irritable bowel syndrome. In randomized testing, ROSE-010 significantly reduced pain intensity compared with placebo (odds ratio 2.30; 95% CI 1.53–3.46), with maximal relief occurring roughly two hours after injection. The greatest benefit occurred in women and in constipation- and mixed-subtype disease [85]. Visceral analgesia is mechanistically coherent with the gut-localized actions emphasized throughout this review (enteroendocrine-nociceptor crosstalk, nitric oxide-dependent effects on visceral sensation, and improved gut-barrier function [38,53]), although ROSE-010 is not commercially available.

### 8.5. Migraine

No trial has directly evaluated GLP-1RAs for migraine. Evidence is limited to a case series reporting reduced headache frequency with tirzepatide, which is confounded by weight loss [86], alongside preclinical data showing that liraglutide attenuated trigeminal allodynia via IL-10 release in a chronic migraine model [87].

### 8.6. Opioid Use and Substance Use Disorders

A recurring and clinically important signal across this literature is a reduced opioid burden. A large cohort study of US veterans with type 2 diabetes (*N* = 524,817) found that GLP-1RA initiation was associated with a reduced risk of incident substance use disorders relative to comparator glucose-lowering agents (HR approximately 0.75) [88]. Among methadone-treated patients with opioid use disorder, GLP-1RA use was associated with higher rates of remission (HR 1.75; 95% CI 1.24–2.47) and lower rates of opioid-related overdose [89]. Preclinical work provides a plausible mechanism at the level of the reward circuitry. GLP-1R activation reduces oxycodone taking and reinstatement of oxycodone seeking without compromising oxycodone’s antinociceptive effect. This acts through GLP-1R on dopamine-receptor-expressing medium spiny neurons in the nucleus accumbens shell [90]. Semaglutide fully attenuates cue-, drug-, and stress-induced fentanyl seeking [91]. That a nutrient-mimetic, microbiota-modulating drug class can simultaneously reduce pain and curb opioid use is a distinctive public-health feature, particularly against the backdrop of the opioid crisis.

**Table 2:** Clinical evidence for GLP-1 receptor agonists in chronic pain conditions.

Pain Condition	Highest-Level Human Evidence	Direction of Effect	Key Limitation
Knee osteoarthritis	Phase 3 RCT (STEP 9, *N* = 407) [68]	Significant pain reduction with semaglutide	Weight-loss contribution hard to isolate; liraglutide trial negative [69]
IIH/headache	Phase 2 RCT (*N* = 15) + meta-analyses [73,75]	↓ intracranial pressure and headache without BMI change	Small RCT; observational data predominate
Diabetic neuropathy	Meta-analysis (*N* = 271) [79]	Improved nerve conduction; mixed pain outcomes	No large RCT with pain as primary endpoint [81]
Fibromyalgia	Propensity-matched cohort [83]	Reduced opioid use and pain diagnoses	Observational; diagnostic-code proxies; no trials
IBS/visceral pain	RCTs of ROSE-010 [84,85]	Significant pain relief	ROSE-010 not commercially available
Migraine	Case series [86]	Suggestive benefit	No trials; confounded by weight loss
Opioid use/OUD	Large cohort [88]	Reduced incident substance use; opioid-sparing	Observational; no pain-specific RCTs

*Abbreviations:* BMI, body mass index; IBS, irritable bowel syndrome; IIH, idiopathic intracranial hypertension; *N*, number of participants; OUD, opioid use disorder; RCT, randomized controlled trial.

## 9. Weight Loss Versus Direct Analgesia

The central interpretive challenge is disentangling weight-dependent from weight-independent analgesia. Several lines of evidence support direct, weight-independent effects: exenatide lowered intracranial pressure and improved headache in idiopathic intracranial hypertension without any change in body mass index [73]. GLP-1RAs improved nerve conduction independent of glucose control [79]. Intrathecal and intracerebroventricular administration produces analgesia at doses far below those that cause metabolic effects [23]. Chondroprotection occurs through a weight-loss-independent mechanism [72], and TRPV1 inhibition is entirely GLP-1R- and metabolism-independent [62]. Against this, the divergence between the positive STEP 9 trial (large weight loss, pain benefit) and the negative liraglutide trial (modest weight loss, no pain benefit) indicates that, at least for musculoskeletal pain, the magnitude of weight loss matters [68,69]. The most parsimonious interpretation is that weight-dependent and weight-independent mechanisms operate in parallel. Their relative contributions vary by pain phenotype; mechanical unloading and metabolic restoration dominate in osteoarthritis, whereas direct neuroimmune and microbial mechanisms dominate in headache, visceral, and neuropathic pain.

## 10. Nutritional and Translational Implications and Future Directions

Viewing GLP-1RAs as nutrient-mimetic, microbiota-modulating agents reframes several translational questions in explicitly nutritional terms. Dietary fiber and fermentable carbohydrates increase endogenous GLP-1 through SCFA-FFAR2 signaling [12,14,15]. Higher fiber intake is also associated with a lower risk of symptomatic knee osteoarthritis in prospective studies [92]. Together, these findings suggest that fiber-rich or prebiotic diets may enhance GLP-1RA analgesia or partially mimic the microbiome changes induced by these drugs. More broadly, nutritional interventions already relieve pain in a majority of chronic musculoskeletal pain studies, plausibly through gut-microbial and anti-inflammatory pathways, positioning diet as a rational adjunct rather than a confounder [5,6]. Beyond fiber alone, whole dietary patterns shape the microbiota in ways that plausibly bear on pain: fermentable fibers, resistant starch, and polyphenol-rich foods expand SCFA-producing and mucin-associated commensals and raise luminal SCFAs, whereas Western-style diets high in saturated fat and refined sugar deplete these taxa and weaken the epithelial barrier [3,47]. Dietary polyphenols in particular have been proposed to restrain chronic inflammation through a gut microbiota-polyphenol-NLRP3 inflammasome axis, converging on the same inflammasome pathway that GLP-1RAs suppress [93]. Clinically, small and mostly uncontrolled studies of prebiotic and fiber-based interventions have reported symptom improvements in visceral and musculoskeletal pain, and observational cohort data associate higher dietary fiber intake with lower incidence and progression of symptomatic knee osteoarthritis [92]; these largely associational findings are hypothesis-generating and are consistent with the view that nutritional strategies and GLP-1RA pharmacology may engage overlapping mechanisms.

This nutritional reframing also clarifies why the weight-loss debate (Section 9) need not be adversarial. Whether analgesia arises from mechanical unloading, from metabolic-inflammatory restoration, or from direct neuroimmune and microbial actions, diet plausibly contributes to all three. A fiber-rich dietary pattern reduces adiposity, enriches SCFA-producing taxa, raises endogenous GLP-1, and lowers systemic inflammation, converging with, rather than competing against, the pharmacology. The clinical implication is that GLP-1RAs and nutritional strategies may be complementary, and that trials evaluating one while ignoring the other may systematically underestimate the achievable effect.

Three priorities follow. First, pain-specific trials should embed longitudinal microbiome and SCFA profiling and, where feasible, dietary standardization, to establish whether microbial change mediates analgesia. Second, molecular engineering that integrates GLP-1R agonism with pain-selective mechanisms, such as TRPV1 inhibition via exendin 20–29, could yield analgesics that retain benefit while shedding metabolic liability [62]. The dual GIP/GLP-1 agonist tirzepatide adds complexity, as GIP receptor signaling is pronociceptive at the spinal level yet analgesic supraspinally [94,95]. Third, safety signals require reconciliation: rapid GLP-1RA-associated weight loss has been linked to diabetic lumbosacral radiculoplexus neuropathy [96]. A large health-outcome mapping reported an association between GLP-1RA use and arthritic disorders, which must be weighed against the osteoarthritis benefit [97]. Semaglutide-specific mechanistic pain data also remain sparse, as most preclinical studies have used exenatide or liraglutide [68].

Finally, the field would benefit from explicit attention to the endogenous end of the gut–brain axis. Most translational efforts have focused on pharmacological GLP-1RAs, yet the same receptor system is physiologically engaged by nutrient- and microbe-driven GLP-1. Interventions that raise endogenous GLP-1 (fermentable fiber, targeted SCFA delivery, or prebiotics) are inexpensive, widely accessible, and carry favorable safety profiles [14,15]. Whether such approaches produce clinically meaningful analgesia on their own, or chiefly potentiate pharmacological therapy, is unknown and eminently testable. Trials that factorially combine dietary and pharmacological arms, with microbiome and SCFA readouts, would directly address the mechanistic claims advanced here and would fit naturally within the scope of nutritional science.

## 11. Strengths and Limitations

Several limitations temper the conclusions drawn here. First, this is a narrative rather than a systematic review; although explicit inclusion and exclusion criteria were applied (Section 2), study selection was not governed by a registered protocol or a formal quantitative risk-of-bias assessment, so selection and reporting biases cannot be excluded. Second, the evidence base is markedly heterogeneous in design and evidentiary weight: it combines rodent mechanistic studies, small early-phase and open-label trials, a single adequately powered randomized controlled trial (STEP 9 in knee osteoarthritis), and several observational or propensity-matched cohorts, which differ substantially in their susceptibility to confounding and in the strength of causal inference they support. Third, much of the mechanistic argument, including the spinal microglial GLP-1R/interleukin-10/beta-endorphin pathway, NLRP3 inflammasome suppression, and microbiota-dependent immunomodulation, rests on preclinical models, and cross-species extrapolation to human pain is assumed rather than demonstrated. Fourth, and most important for the central thesis, no human study has yet shown that GLP-1RA-induced changes in the gut microbiota causally mediate analgesia; the microbiota-as-intermediary model is therefore a mechanistically plausible hypothesis that remains to be tested directly in patients. Fifth, weight loss is a pervasive potential confounder that is difficult to disentangle fully from weight-independent effects in the available clinical datasets. Finally, pain outcomes were heterogeneously defined across studies, and few trials were designed with pain as a primary endpoint. The principal strength of this review is its integration of nutrient-sensing physiology, microbial signaling, and neuroimmune pharmacology into a single, testable framework; its principal limitation is that this framework currently outpaces the direct human evidence, underscoring the need for the dedicated, mechanistically instrumented trials outlined in Section 10.

## 12. Conclusions

GLP-1 sits at a nutritional crossroads. Enteroendocrine L-cells secrete it in response to nutrients and microbial short-chain fatty acids, which then signals pain circuits through vagal, humoral, and neuroimmune pathways. Its pharmacological agonists modulate chronic pain through at least ten mechanisms spanning the pain neuraxis, most consistently, in preclinical models, the spinal microglial GLP-1R/IL-10/beta-endorphin pathway, and they do so in large part independently of glycemic control and weight loss. Crucially, GLP-1RAs enrich the same short-chain fatty acid-producing, anti-inflammatory taxa that are depleted in chronic pain, and they require an intact microbiota for their full anti-inflammatory effect. The gut microbiota is therefore proposed to act as both a therapeutic target and a mechanistic intermediary linking GLP-1RA pharmacology to analgesia. It must be emphasized, however, that much of this mechanistic evidence derives from rodent models and small early-phase studies, and that direct human data linking microbiota-dependent or neuroimmune mechanisms to clinical pain relief remain limited. Clinical evidence is strongest for knee osteoarthritis and idiopathic intracranial hypertension headache, with only preliminary signals in fibromyalgia, visceral pain, and opioid-use reduction. Confirming these mechanisms will require pain-specific trials that treat diet and the microbiome as central, measurable components rather than nuisance variables.

## Figures and Tables

**Figure 1:**
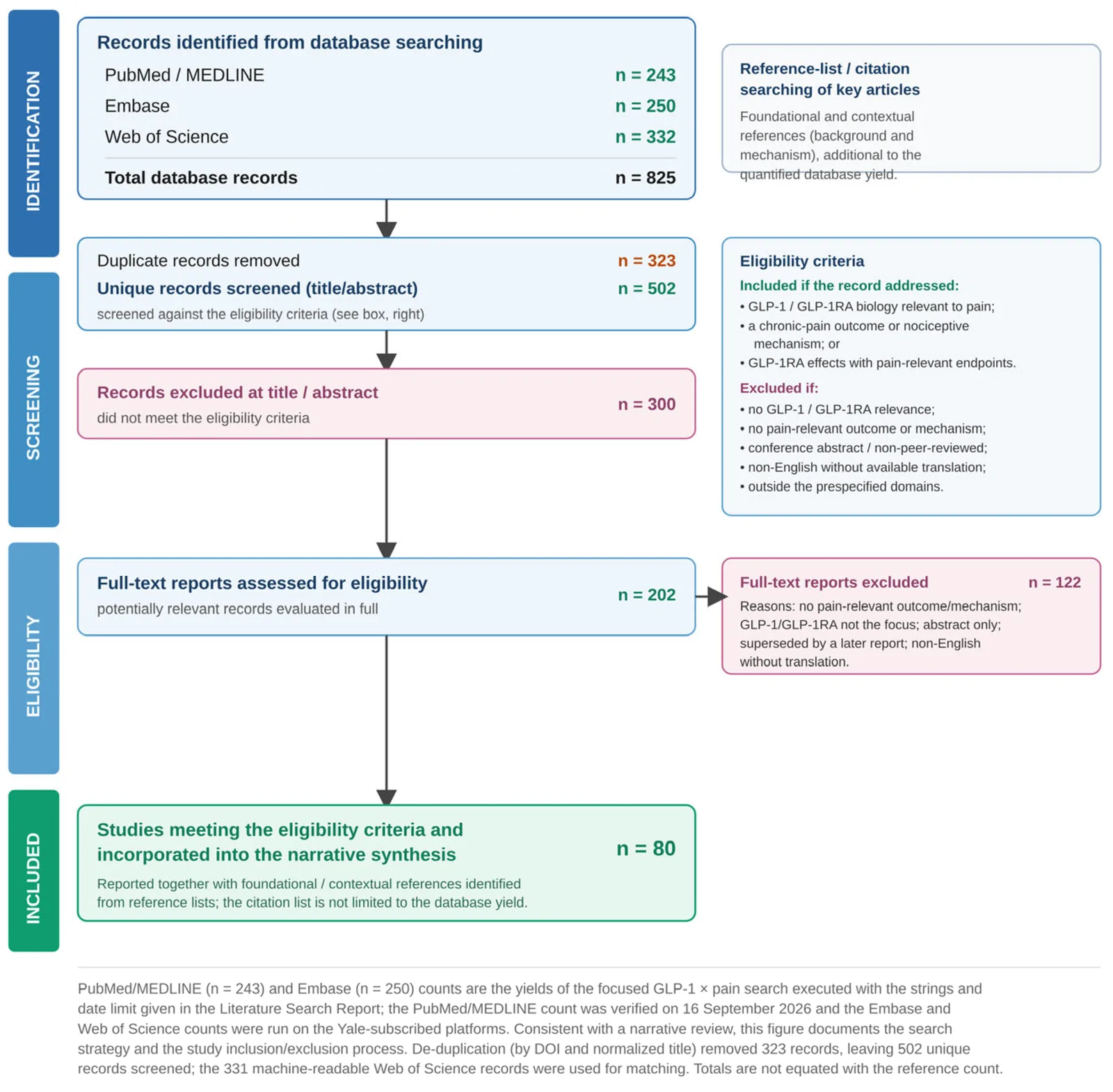
Literature search and study-selection flow. A focused search combining GLP-1 terms with pain terms (databases searched from inception to 30 June 2026) identified 825 records—PubMed/MEDLINE [*n* = 243], Embase [*n* = 250], and Web of Science [*n* = 332]. After removal of 323 duplicate records, the remaining 502 unique records were screened by title and abstract, of which 300 were excluded; the 202 full-text reports assessed for eligibility yielded 80 studies that met the criteria (122 excluded at full text, with reasons shown). Foundational and contextual references were additionally identified from the reference lists of key articles. The full per-database search strings and criteria are compiled in File S1.

**Figure 2:**
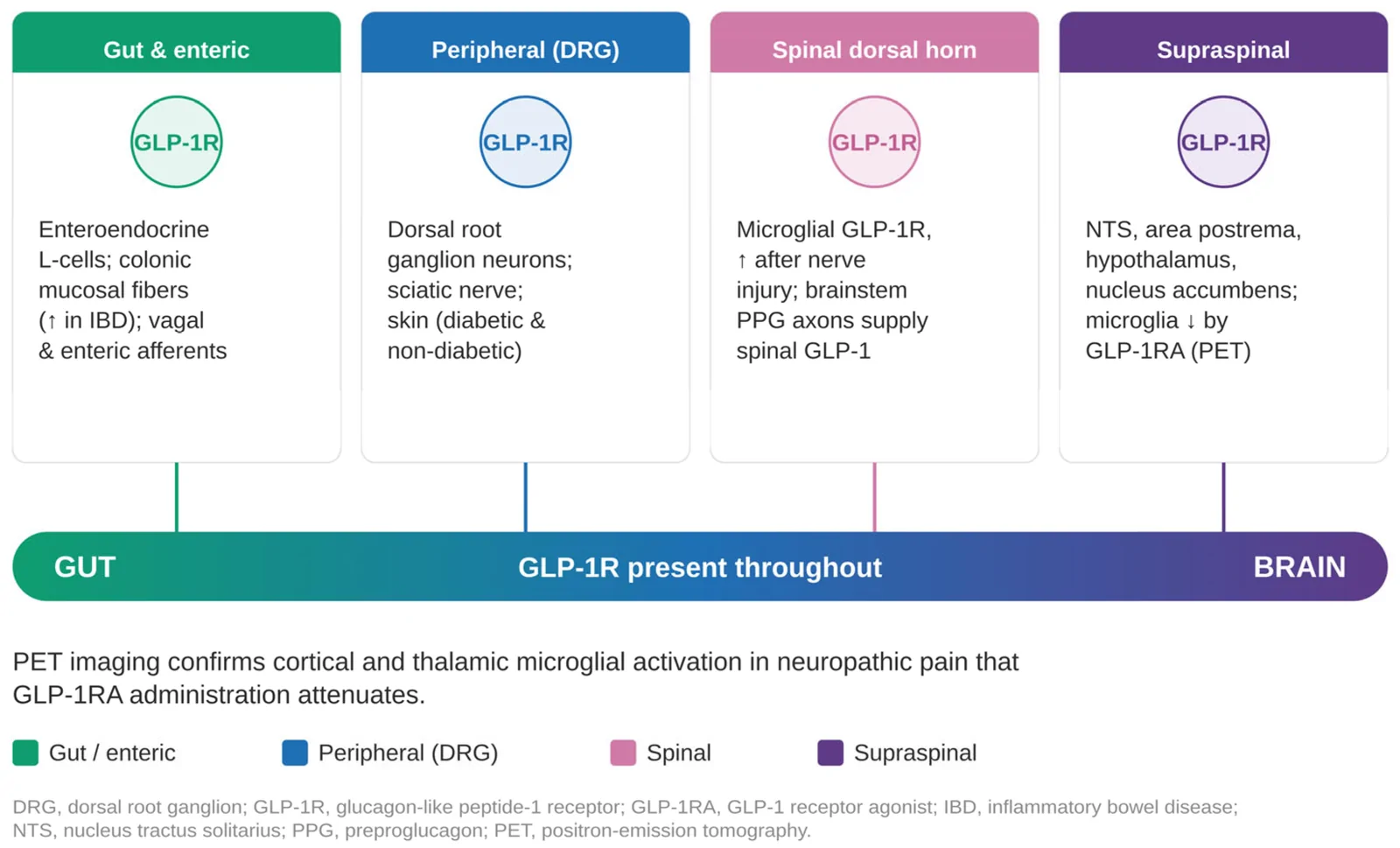
GLP-1 receptor expression along the gut-pain neuraxis, from colonic mucosal and vagal afferents through spinal dorsal horn microglia to supraspinal pain centers.

**Figure 3:**
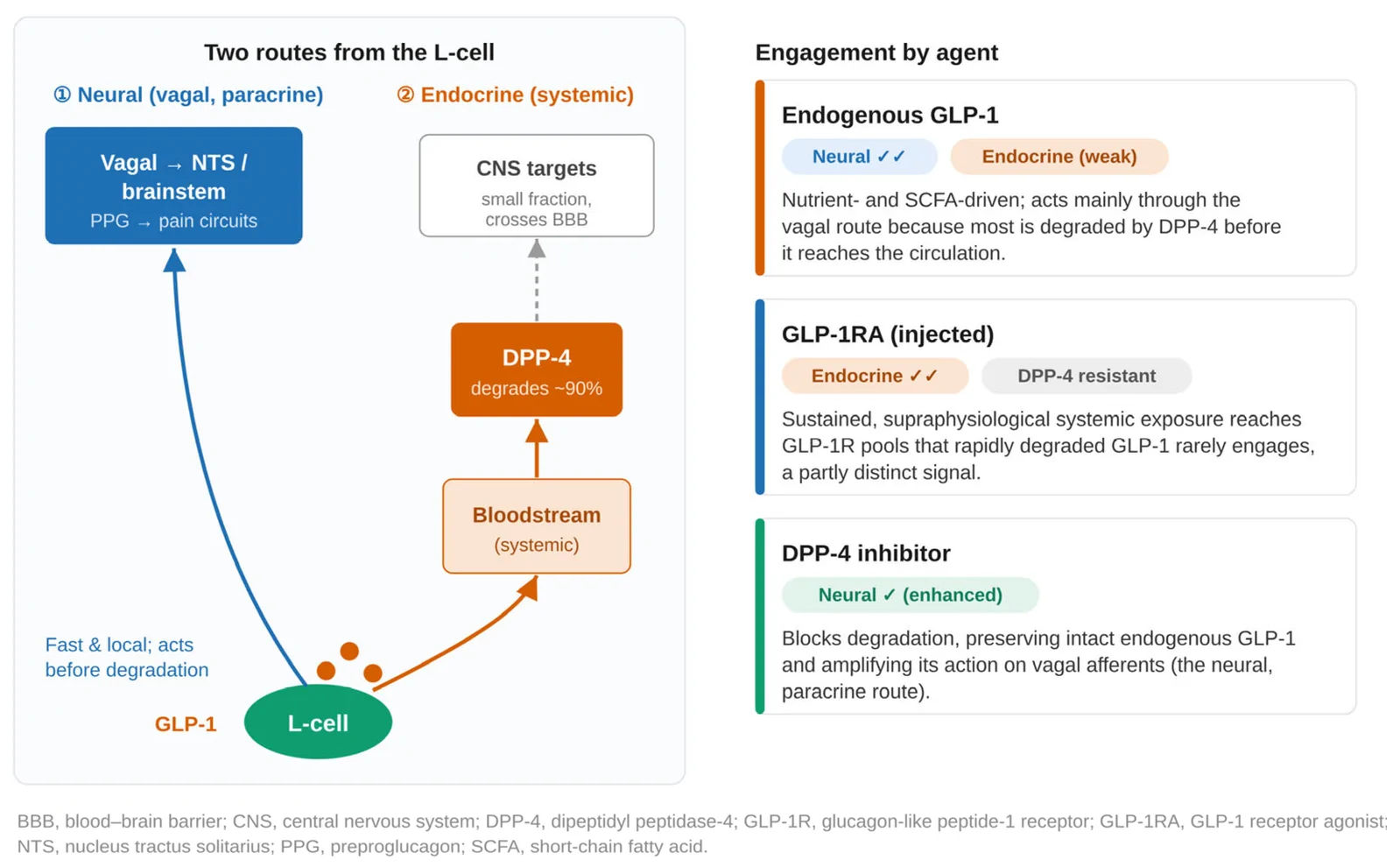
Endocrine versus neural routes of GLP-1 action, and the differential engagement of these routes by endogenous GLP-1, GLP-1 receptor agonists, and DPP-4 inhibitors.

**Figure 4:**
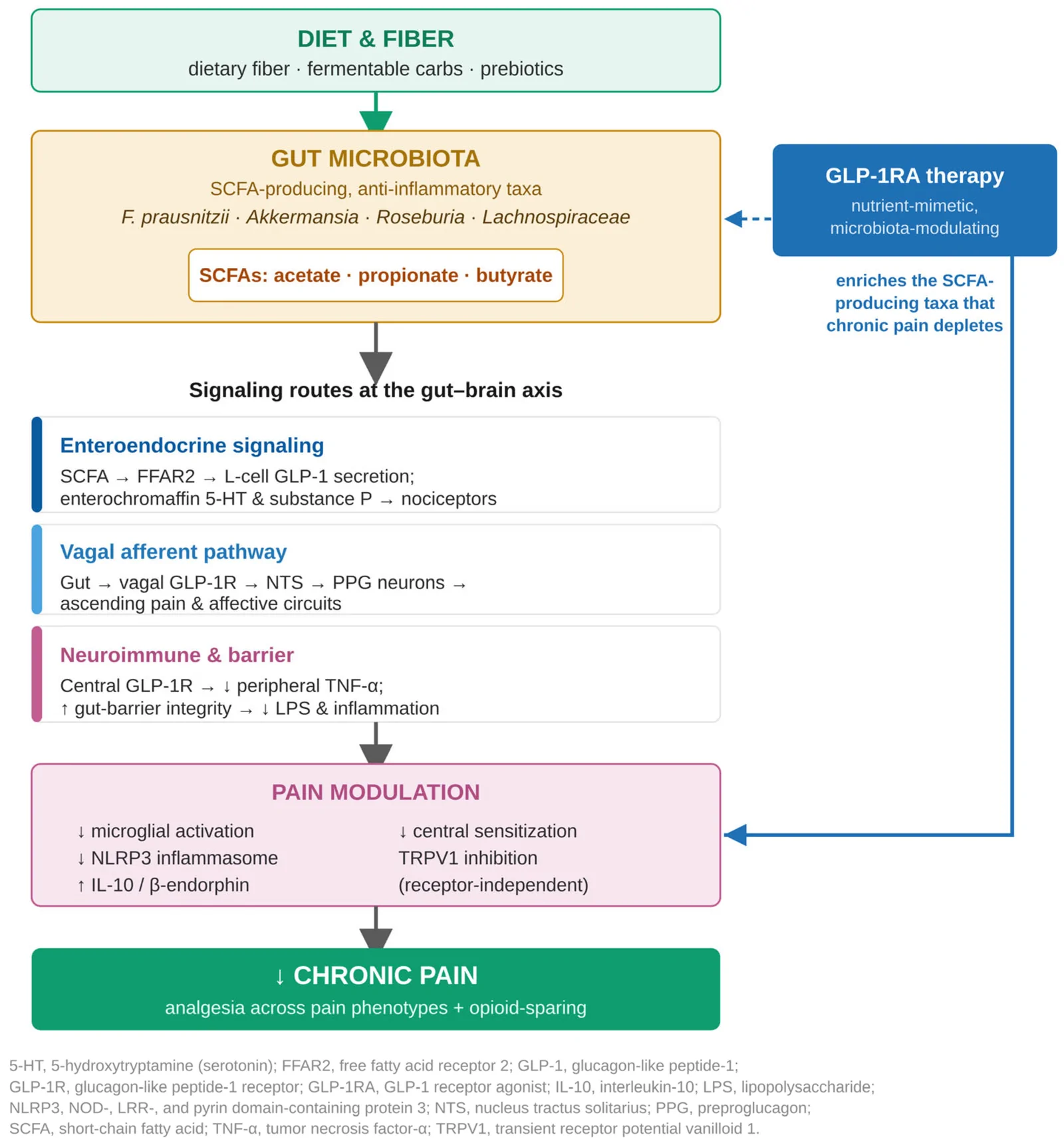
Integrative model: dietary fiber and microbial short-chain fatty acids drive enteroendocrine, vagal, and neuroimmune signaling that converges with GLP-1 receptor agonist pharmacology to modulate pain circuits at the gut–brain axis.

**Figure 5:**
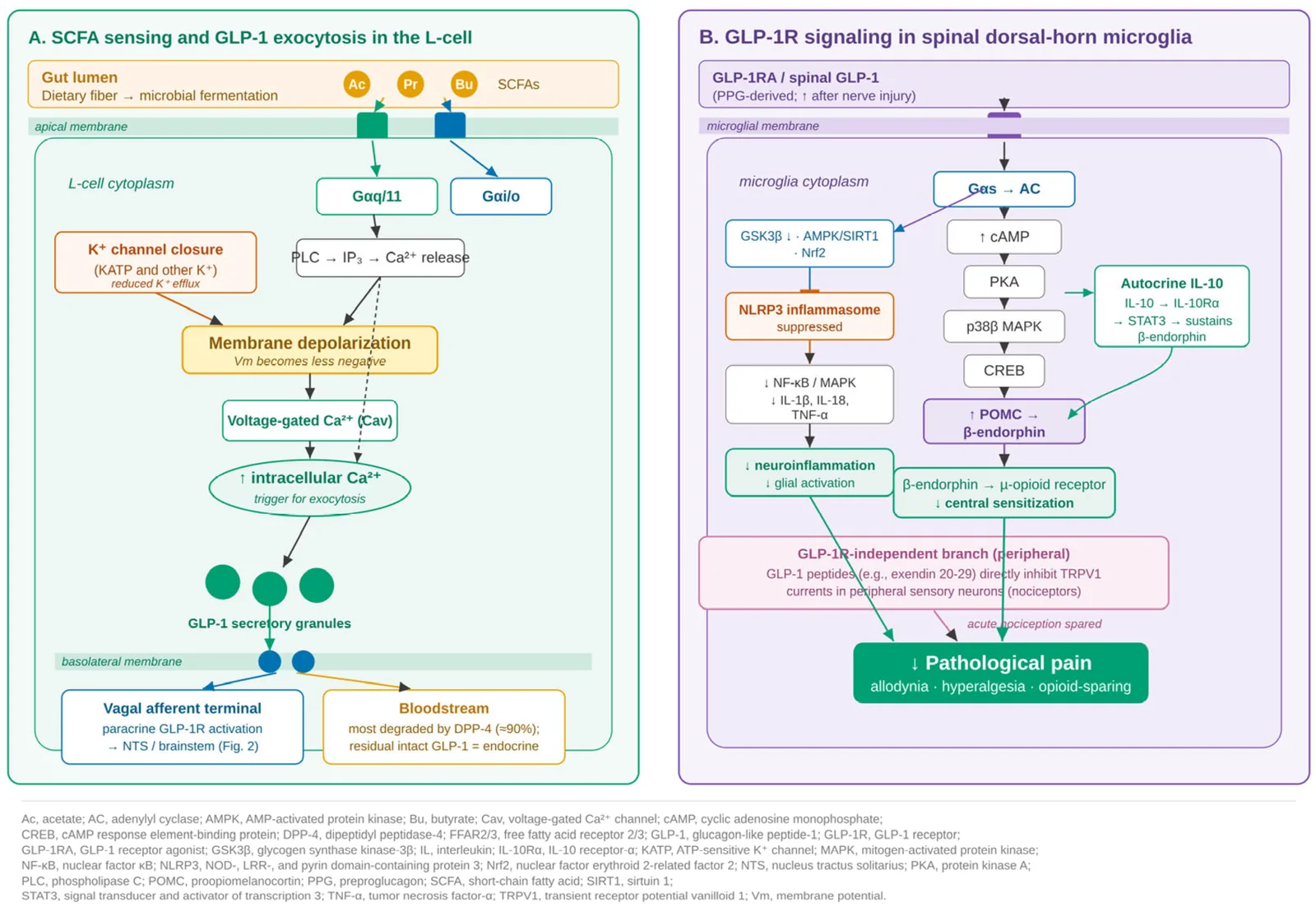
Molecular mechanisms linking short-chain fatty acid sensing to GLP-1 receptor-mediated analgesia. (**A**) In the enteroendocrine L-cell, short-chain fatty acids (SCFAs) engage the free fatty acid receptors FFAR2 and FFAR3, which couple through Gq/11 and Gi/o proteins to raise intracellular Ca2+ and, together with closure of ATP-sensitive and other K+ channels, depolarize the plasma membrane; the resulting voltage-gated Ca2+ entry triggers Ca2+-dependent exocytosis of GLP-1-containing secretory granules, which are released onto adjacent vagal afferents (paracrine route) and into the bloodstream (endocrine route, where most GLP-1 is rapidly degraded by dipeptidyl peptidase-4). (**B**) In spinal dorsal-horn microglia, GLP-1 receptor (GLP-1R) activation signals through Gs/adenylyl cyclase/cAMP/protein kinase A/p38beta mitogen-activated protein kinase/cAMP response element-binding protein to drive proopiomelanocortin-derived beta-endorphin expression, reinforced by an autocrine interleukin-10/STAT3 loop; beta-endorphin then acts on mu-opioid receptors to reduce central sensitization. In parallel, GLP-1R signaling suppresses the NLRP3 inflammasome (via GSK3beta, AMPK/SIRT1, and Nrf2) and NF-kB/mitogen-activated protein kinase-driven cytokine production, whereas GLP-1-derived peptides inhibit TRPV1 on peripheral sensory neurons independently of GLP-1R. These actions converge on reduced pathological pain (allodynia and hyperalgesia) while sparing acute nociception. Abbreviations are defined within the figure.

## Data Availability

No new data were created or analyzed in this study. Data sharing does not apply to this article.

## Supplementary Materials

The following supporting information can be downloaded at https://www.mdpi.com/article/10.3390/nu18193120/s1, File S1: Literature Search Strategy and Study Selection (full per-database search strings, record counts, eligibility criteria, and the PRISMA-style study-selection flow).
