# Supplementary material for: GLP-1 Receptor Agonists, Gut Microbiota, and Chronic Pain: Nutritional Signaling at the Gut–Brain Axis
Source: Nutrients. 2026 Sep 22;18(19):3120. doi: 10.3390/nu18193120 (PMC13667044; doi:10.3390/nu18193120)
Supplement: Supplementary file 1 [file nutrients-18-03120-s001.zip › nutrients-4534232-supplementary.pdf]

Supplementary Material S1

Manuscript nutrients-4534232

GLP-1 Receptor Agonists, Gut Microbiota, and Chronic Pain: Nutritional Signaling at the Gut–Brain Axis

Literature Search Strategy and Study Selection

Summary. Three databases (PubMed/MEDLINE, Embase, Web of Science) were searched from inception to 30 June 2026 with a focused GLP-1 × pain strategy: 825 records identified [PubMed/MEDLINE n = 243; Embase n = 250; Web of Science n = 332]; 323 duplicates removed; 502 unique records screened by title/abstract (300 excluded); 202 full-text reports assessed for eligibility, of which 122 were excluded and 80 studies met the prespecified eligibility criteria below and were incorporated into the narrative synthesis (Figure 1). The PubMed/MEDLINE count was verified 16 September 2026; Embase and Web of Science were run on the Yale-subscribed platforms.

This Supplementary Material documents the literature search and study-selection process for the narrative review, prepared in response to Reviewer 2. It records the databases searched, the exact search strings, the date limit, the number of records identified in each database, the eligibility criteria, and the study-selection flow, which appears in the main text as Figure 1 and is reproduced here. Consistent with a narrative review, it documents the search strategy and how studies were included or excluded; it does not equate the database yield with the manuscript's reference count, which also includes foundational and contextual works.

1. Search narrative

The eligibility search combined a GLP-1 / incretin concept block ("GLP-1," "glucagon-like peptide-1," "incretin," "GLP-1 receptor agonist," and the agents semaglutide, liraglutide, exenatide, tirzepatide, and dulaglutide) with a pain concept block ("chronic pain," "nociception," "neuropathic pain," "osteoarthritis," "fibromyalgia," "visceral pain") using the Boolean AND, with field tags adapted to each platform. PubMed/MEDLINE, Embase (OVID SP), and Web of Science (Core Collection) were searched from database inception through 30 June 2026. This focused search defines the pool of potentially eligible pain-related studies and is quantified in the selection flow (Figure 1).

To inform the mechanistic and physiological background of the review, we additionally ran broader scoping searches combining the GLP-1 block with gut-microbiota and short-chain-fatty-acid terms ("gut microbiota," "gut microbiome," "short-chain fatty acids," "enteroendocrine," "gut-brain axis"), and we hand-searched the reference lists of key reviews and primary studies. Because the objective was integrative narrative synthesis rather than a pooled treatment estimate, no quantitative meta-analysis or formal study-level risk-of-bias assessment was performed; potentially relevant records were screened by title and abstract against the eligibility criteria (Section 3) and assessed in full text.

2. Databases, date limit, and record counts

Database	Exact search string (GLP-1 AND pain) and date limit	Records (n)
**PubMed / MEDLINE** (verified 16 Sep 2026)	`(GLP-1[tiab] OR "glucagon-like peptide-1"[tiab] OR "glucagon like peptide 1"[tiab] OR incretin[tiab] OR "GLP-1 receptor agonist"[tiab] OR GLP-1RA[tiab] OR semaglutide[tiab] OR liraglutide[tiab] OR exenatide[tiab] OR tirzepatide[tiab] OR dulaglutide[tiab]) AND ("chronic pain"[tiab] OR nociception[tiab] OR "neuropathic pain"[tiab] OR osteoarthritis[tiab] OR fibromyalgia[tiab] OR "visceral pain"[tiab])` Date filter: 1900/01/01 : 2026/06/30 [Date - Publication].	**243**
**Embase (OVID SP)**	`(glucagon-like peptide 1 or GLP-1 or GLP1 or incretin or GLP-1 receptor agonist or semaglutide or liraglutide or exenatide or tirzepatide or dulaglutide).ti,ab,kw. AND (chronic pain or nociception`	**250**

Database	Exact search string (GLP-1 AND pain) and date limit	Records (n)
	`or neuropathic pain or osteoarthritis or fibromyalgia or visceral pain).ti,ab,kw.` Limited to publication date ≤ 30 June 2026.	
**Web of Science** (Core Collection)	`TS=("GLP-1" OR "glucagon-like peptide-1" OR incretin OR "GLP-1 receptor agonist" OR semaglutide OR liraglutide OR exenatide OR tirzepatide OR dulaglutide) AND TS=("chronic pain" OR nociception OR "neuropathic pain" OR osteoarthritis OR fibromyalgia OR "visceral pain")` Timespan to 30 June 2026.	**332**
**Total (databases)**	Sum across the three databases before de-duplication.	**825**
**Hand-searching**	Reference lists of key reviews and primary studies screened for seminal or otherwise relevant records not captured by the database queries (used chiefly for background and mechanism).	—

Background scoping searches (not part of the eligibility count; PubMed/MEDLINE, same date limit, for transparency): **GLP-1 AND gut-microbiota terms = 2,191; GLP-1 AND microbiota AND pain = 11.** These informed the physiological and microbiological background sections and were supplemented by reference-list searching.

3. Eligibility criteria

Included if the record addressed one or more of:

- GLP-1 or GLP-1 receptor agonist (GLP-1RA) biology relevant to pain;
- a chronic-pain outcome or a nociceptive mechanism;
- GLP-1RA effects with pain-relevant endpoints (clinical or preclinical).
- Eligible designs: mechanistic in vitro and animal studies, human translational studies, randomized controlled trials, observational/propensity-matched cohorts, and systematic reviews or meta-analyses.

Excluded if:

- no GLP-1 / GLP-1RA relevance;
- no pain-relevant outcome or mechanism;
- conference abstract or other non-peer-reviewed report;
- non-English publication without an available translation;
- outside the prespecified conceptual domains.

4. Study-selection flow (PRISMA-style)

The study-selection flow (the manuscript's Figure 1, reproduced below) summarizes identification, screening, eligibility, and inclusion. The three database counts (PubMed/MEDLINE 243, Embase 250, Web of Science 332; 825 total before de-duplication) are the verified yields of the focused GLP-1 × pain search. After de-duplication by DOI and normalized title, 323 duplicate records were removed, leaving 502 unique records for title/abstract screening against the eligibility criteria. Of these, 300 records were excluded at the title/abstract stage; the 202 full-text reports subsequently assessed for eligibility yielded 80 studies that met the criteria (122 excluded at full text), which were incorporated into the narrative synthesis. The figure documents the search and selection process; it does not tie the database yield to the 97-item reference list, which also contains foundational and contextual citations identified from reference lists.

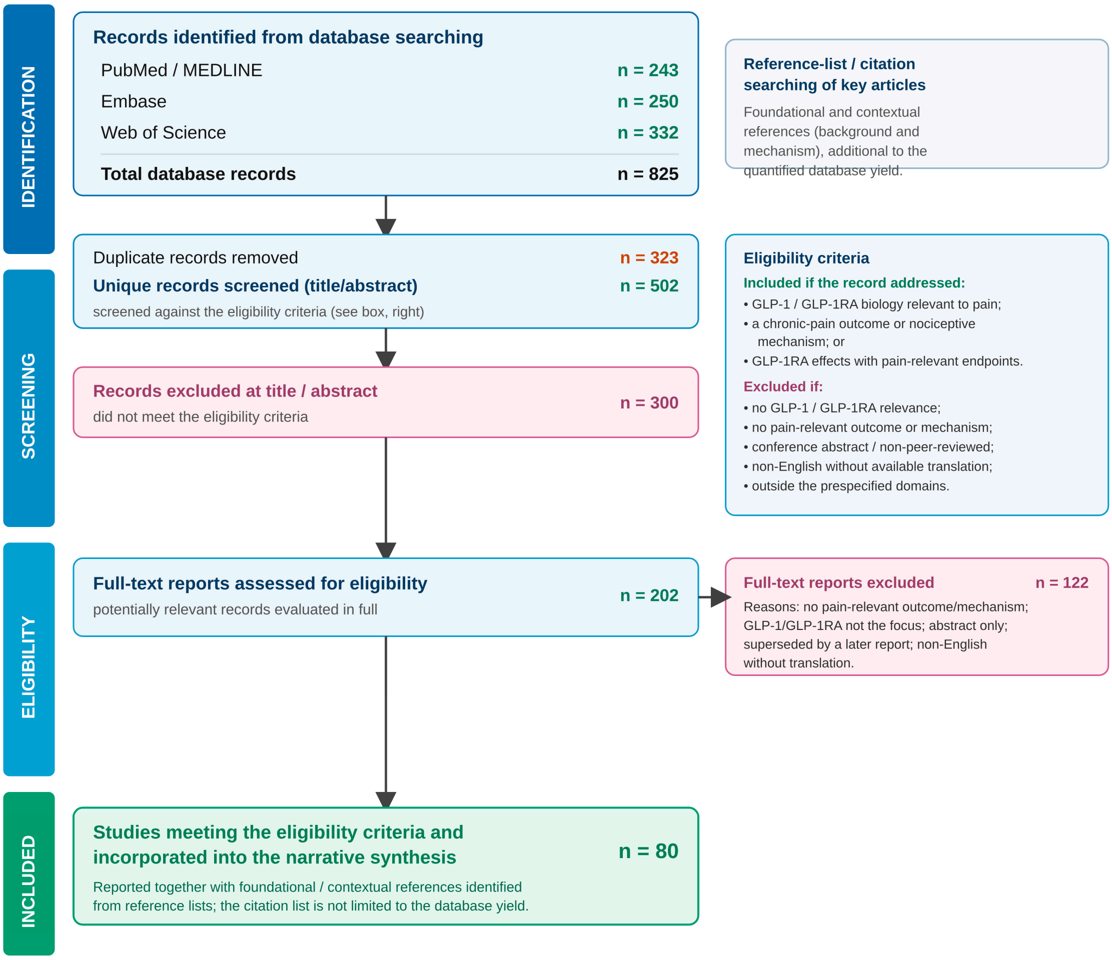

PubMed/MEDLINE (n = 243) and Embase (n = 250) counts are the yields of the focused GLP-1 × pain search executed with the strings and date limit given in the Literature Search Report; the PubMed/MEDLINE count was verified on 16 September 2026 and the Embase and Web of Science counts were run on the Yale-subscribed platforms. Consistent with a narrative review, this figure documents the search strategy and the study inclusion/exclusion process. De-duplication (by DOI and normalized title) removed 323 records, leaving 502 unique records screened; the 331 machine-readable Web of Science records were used for matching. Totals are not equated with the reference count.

5. Reproducibility note

The three searches are reproducible: pasting each Section 2 string into the corresponding platform with the stated date limit returns the reported count (the count is fixed by the ≤ 30 June 2026 publication-date limit; live totals grow as records accrue and as databases re-index). The PubMed/MEDLINE search was executed and verified on 16 September 2026; the Embase (OVID SP) and Web of Science (Core Collection) searches were run on the Yale-subscribed platforms. De-duplication across databases and the title/abstract and full-text screening were performed by the review team against the Section 3 criteria.
